# American Tegumentary Leishmaniasis: Effectiveness of an Immunohistochemical Protocol for the Detection of *Leishmania* in Skin

**DOI:** 10.1371/journal.pone.0063343

**Published:** 2013-05-21

**Authors:** Cibele Fontes Alves, Cintia Fontes Alves, Maria Marta Figueiredo, Carolina Carvalho Souza, George Luiz Lins Machado-Coelho, Maria Norma Melo, Washington Luiz Tafuri, Pedro Raso, Rodrigo Pedro Soares, Wagner Luiz Tafuri

**Affiliations:** 1 Departamento de Patologia Geral, Instituto de Ciências Biológicas, Universidade Federal de Minas Gerais, Belo Horizonte, Minas Gerais, Brasil; 2 Departamento de Parasitologia, Instituto de Ciências Biológicas, Universidade Federal de Minas Gerais, Belo Horizonte, Minas Gerais, Brasil; 3 Departamento de Ciências Médicas, Escola de Farmácia, Universidade Federal de Ouro Preto, Ouro Preto, Minas Gerais, Brasil; 4 Departamento de Anatomia Patológica, Faculdade de Medicina, Universidade Federal de Minas Gerais, Belo Horizonte, Minas Gerais, Brasil; 5 Centro de Pesquisas René Rachou/FIOCRUZ, Belo Horizonte, Minas Gerais, Brasil; Blood Systems Research Institute, United States of America

## Abstract

**Background:**

American tegumentary leishmaniasis (ATL) is endemic in Latin America, where Brazil has over 27 thousand cases per year. The aim of the present study was to develop an immunohistochemical method (IHC) for ATL diagnosis. For this purpose, we used serum from a dog naturally infected with *Leishmania* (*L*) *infantum* (canine hyperimmune serum) as the primary antibody, followed by a detection system with a secondary biotinylated antibody.

**Methodology:**

Skin samples were obtained from 73 patients in an endemic area of Caratinga, Minas Gerais (MG) State, Brazil all testing positive for ATL with the Montenegro skin test, microscopy, and PCR. Canine hyperimmune serum of a dog naturally infected with *Leishmania* (*L.*) *infantum* was employed as a primary antibody in an immunohistochemical diagnostic method using streptavidin-biotin peroxidase. To assess the specificity of this reaction, IHC assays employing two monoclonal antibodies were carried out. As the polymer-based technology is less time-consuming and labor intensive than the IHC labeled streptavidin-biotin peroxidase method, we compared the two methods for all samples.

**Results:**

The IHC method detected ATL in 67 of the 73 cases (91.8%). Immunolabeled parasites were primarily detected inside macrophages either in the superficial or the deep dermis. Detection was facilitated by the high contrast staining of amastigotes (dark brown) against the light blue background. A lower detection rate (71.2%) was observed with the both of the monoclonal *Leishmania* antibodies compared to the canine hyperimmune serum. This may have been due to a non-specific background staining observed in all histological samples rendering positive detection more difficult. The higher efficacy of the canine hyperimmune serum in the IHC method was confirmed by the method using streptavidin-biotin peroxidase as well as that with the polymer-based technology (biotin-avidin-free system).

**Conclusions:**

The data are encouraging with regard to validating IHC as a standard alternative method for ATL diagnosis.

## Introduction

Leishmaniasis is a tropical and neotropical disease caused by more than 20 species of *Leishmania*. In the New World, as a zoonotic disease, the transmission of these parasites to humans occurs through the bite of the sandfly vector [1]. Leishmaniasis has been considered among the most neglected of diseases because of the limited resources invested in its diagnosis, treatment, and control, along with its strong association with poverty and social conflicts [2]–[4]. It ranges from localized skin ulcers called cutaneous leishmaniasis (CL) to a lethal systemic disease, visceral leishmaniasis (VL).Cutaneous leishmaniasis is endemic in more than 70 countries, with 90% of cases occurring in Afghanistan, Algeria, Brazil, Pakistan, Peru, Saudi Arabia, and Syria. In Brazil, the disease is also known as American tegumentary leishmaniasis (ATL) caused by *Leishmania* (*Viannia*) *braziliensis, Leishmania* (*Viannia*) *guyanensis,* and *Leishmania* (*Leishmania*) *amazonensis.* However, the majority of cases are caused by *L.* (*V*.) *braziliensis,* producing the principal clinical form of ATL: localized cutaneous leishmaniasis (LCL). The earliest records of ATL in Minas Gerais, Brazil, are related to deforestation for road construction and agriculture activities. Transmission of ATL in Minas Gerais, as in other Brazilian regions, has changed in recent years, with outbreaks occurring in long-established rural settlements and urban areas. The cutaneous disease is reported to occur with greater frequency in economically deprived areas lacking essential sanitation, with low education levels and low income [4], [5].

The most frequent tests for ATL diagnosis are the Montenegro skin test (MST) and direct detection of the parasite in lesions (histological staining of tissue sections, impression smears of punch biopsies followed by Giemsa staining and smears of dermal scraping from the lesion margins). Serological and molecular approaches have been carried out, but parasite recognition remains the gold standard for CL because of its lack of ambiguity. It comprises the microscopic examination of Giemsa-stained biopsy smears or aspirate, histopathological examination of fixed skin lesion biopsies, immunocytochemical and immunohistochemical (IHC) approaches, and the culture of biopsy triturates or aspirates [6]–[8]. Microscopic examination is probably the most common diagnostic method, because more sophisticated techniques are expensive and not widely available in the endemic areas of Brazil. Although histopathology of lesions is frequently requested by physicians, the sensitivity of this method is highly variable (14–50%), and is even lower for MCL [9].

Immunohistochemistry has become one of the most important techniques in the characterization of many human diseases, including leishmaniasis [10]–[13]. A distinguishing feature of IHC, compared to other diagnostic tests, is its ability to identify an antigen *in situ* in normal or affected tissues. Our aim was to investigate its potential for ATL diagnosis in humans using a dog hyperimmune serum as the primary antibody, followed by detection using two distinct systems with secondary biotinylated antibodies.

## Materials and Methods

### Ethics Statement

This trial was conducted according to the Declaration of Helsinki principles. Prior to enrolment in the study, all patients received a written copy of the study policy, which was reviewed with them individually by an independent person. Written informed consent was obtained from all adult patients and from the parents or guardians of minors. The study was approved by the Ethics Committee of the Federal University of Minas Gerais (COEP/UFMG-Par/Res 211/2011), Belo Horizonte, Brazil, as in compliance with the Declaration of Helsinki of the World Medical Association (WMA, 2008 in 59^th^WMA General Assembly, Seoul, Korea, October 2008).

Procedures for the dog hyperimmune serum protocol were approved by the CETEA/UFMG (Comitê de Ética em Experimentação Animal/Universidade Federal de Minas Gerais), protocol 187/2011. All procedures involving animals were conducted according to the guidelines of the Colégio Brasileiro de Experimentação Animal (COBEA).

### American Tegumentary Leishmaniasis (ALT) Endemic Area

All patients live in the Caratinga area, Minas Gerais (MG) State, where ATL is endemic. This area is located in southeastern Brazil, within the boundaries of 19° 19′ and 20° 01′ S and 41° 46′ and 42° 31′ W, an area of 2,234 km^2^, located in the Rio Doce Valley, 575 m above sea level. *Leishmania (V.) braziliensis* has been identified as the sole causal agentof leishmaniasis in this area in recent years [5], [7]. Patients attended the Dr. Paulo Araújo Magalhães referral clinic in the municipality of Caratinga, MG, which is solely concerned with the diagnosis and treatment of ATL and works in collaboration with the Leishmaniasis Section, Parasitology Department, Federal University of Minas Gerais (UFMG), Belo Horizonte.

### Patient Selection

All patients included in the study had a confirmed diagnosis of ATL identified clinically as localized cutaneous leishmaniasis (LCL). Tissue smears were positive for ATL. Then, all patients previously diagnosed by ATL were selected for inclusion based on the following criteria: gender, age, positive Montenegro skin test (MST), and no previous treatment with anti-*Leishmania* drugs. All subjects were biopsied for immunohistochemical and histopathological analysis and polymerase chain reaction (PCR). Patient identification data (age, gender, and home location), year of first medical attendance, time between onset of lesions and initial consultation, clinical characteristics, and number and sites of lesions were obtained by analyzing the medical records of patients for the diagnosis and treatment of leishmaniasis in Caratinga/MG.

### Montenegro Skin Test

The *Leishmania* antigen used for MST was obtained from the *L. (L.) amazonensis* strain containing 40 µg of proteic nitrogen/ml. The magnitude of the skin response was assessed 48–72 h after intradermal injection of 0.1 ml of the antigen in the anterior of the right forearm [14], [15]. The diameter of the induration was measured in millimeters by outlining the indurated border, with MST considered positive if it measured ≥5 mm [16].

### Parasitological Diagnosis of *Leishmania* Infection

Incisional skin biopsy specimens were taken from the borders of lesions of each of the 73 patients enrolled in the study. Before fixing in methanol, a tissue imprint was made and stained with Giemsa for microscopic examination. *Leishmania* amastigotes were detected by light microscopy using oil immersion (×1000 magnification). The infection was confirmed by PCR as *Leishmania (V.) braziliensis* without co-infection *with Leishmania* (*L.*) *infantum* (*syn L.chagasi*) [17], [18] infection. DNA extraction from skin fragments embedded in paraffin followed the protocol of the “NucleoSpin ® Tissue (Macherey-Nagel) kit with modifications as follows: PCR conditions used 1 ng of DNA. Positive control used DNA from a *L. braziliensis* culture (MHOM/BR/75/M2904). Negative controls used DNA extracted from uninfected skin. PCR primers, conditions, and thermal profiles were as described. The PCR amplified products were resolved in non-denaturing 5% polyacrylamide gel and silver stained. Primers were used and amplified a 90 bp fragment of a single-copy-number gene of DNA polymerase of *Leishmania (L.) infantum* (GenBank accession number AF009147) [19].

### Histopathology

A 3 mm skin biopsy was taken from the edge of the lesions with a sterile surgical blade using 2% xylocaine as anesthetic. Ulcerated and crusted areas of the lesion were avoided. All skin biopsies of patients with LCL, which would routinely be discarded after completion of the smear, were retained and assessed histologically and with immunohistochemistry. Samples were fixed in 10% neutral buffered formalin (pH 7.2) for at least 78 h, then routinely processed and sectioned at 3–4 µm and stained with hematoxylin and eosin (HE).

Chronic inflammatory reactions in samples were based of the presence or absence of plasma cells, macrophages (epithelioid cells and giant cells), lymphocytes, unorganized or organized granulomas, necrosis, and a low frequency of polymorphonuclears (neutrophils and eosinophils) in stained slides. Cells were assessed using a semi-quantitative procedure (slight to intense). Hematoxylin and eosin staining was also used to characterize amastigote forms of *Leishmania* according to their size, shape, and location inside macrophages, and to estimate parasite numbers using the semi-quantitative procedure. The scoring system was based on previously report [20], [21] as follow: 1 = absent, no mononuclear cell exudate (apparently histologically normal dermis); 2 = slight, diffuse mononuclear exudate in the upper dermis (1–9 cells per field/20 fields); 3 = moderate, a diffuse or focal mononuclear exudate around the vessels, glands, and hair follicles in the deep dermis or hypodermis (10–30 cells per field/20 fields); and 4 = intense, a severe diffuse or focal mononuclear exudate around the vessels, glands, and hair follicles in deep dermis or hypodermis (>30 cells per field/20 fields).

### Collection of Hyperimmune Dog Serum

A mixed-breed adult dog of unknown age was obtained from the Control Zoonosis Center of the Municipality of Ribeirão das Neves, Belo Horizonte Metropolitan area, Minas Gerais, Brazil. It was diagnosed with *Leishmania* (*L.*) *infantum* infection serologically and parasitologically [22]. The indirect immunofluorescence antibody test (IFAT) titer was >1∶40 dilution and the enzyme linked immunosorbent assay (ELISA) showed optical density >100 (1∶400 dilution). PCR was conducted on liver and spleen samples (samples tissue obtained from euthanasia of the infected animal) to confirm the visceral infection by *Leishmania* (*L*.) *infantum.* No *Leishmania (V.) braziliensis* co-infection was found by PCR [22].Ten ml of peripheral blood samples were collected by jugular venipuncture after trichotomy and local antisepsis into 10 ml disposable sterile syringes with 21G1 needle (0.80 mm×25 mm) to obtain the hyperimmune serum. Aliquots of 20 µl of this serum were maintained at −20°C.

### Labeling Amastigotes of *Leishmania* Using Streptavidin-biotin Peroxidase IHC with Dog Hyperimmune Serum as the Primary Antibody

The streptavidin-biotin immunohistochemical method was standardized, as previously reported [23] for human tissue, with ATL. Paraffinized skin biopsies were exposed to differing concentrations of hyperimmune serum (primary antibody) from a dog naturally infected with *Leishmania* (*L*.) *infantum*. Dilutions of the dog serum were 1∶100 was found to be most effective in all cases. The stability of the dog serum primary antibody was confirmed according to the criteria reported [6]. Deparaffinized slides of human skin samples were hydrated and incubated in 4% hydrogen peroxide in 0.01 M PBS, pH 7.2, to block endogenous peroxidase activity, followed by incubation with goat serum (1∶100 dilution) and/or Molico® milk powder to block nonspecific immunoglobulin binding. The hyperimmune serum (IFAT; titers ≥ 1∶40) used as the primary antibody was diluted at 1∶10 to 1∶2000 in 0.01 M PBS. Slides were incubated with primary antibody for 18 to 22 h at 4°C in a humid chamber. After washing in PBS, the slides were incubated with biotinylated goat anti-mouse and anti-rabbit (Link-DAKO, LSAB2 Kit, Catalog # KO675-1; Carpinteria, California, USA), washed in PBS again, and incubated with the streptavidin-peroxidase complex (DAKO, LSAB2 Kit, Catalog # K0675-1; Carpinteria, California, USA) for 20 min at room temperature. The reaction was highlighted with a 0.024% diaminobenzidine (DAB; Sigma, St. Louis, USA) solution (chromogen) and 0.16% hydrogen peroxide. Finally, slides were dehydrated, cleared, counter-stained with Harris’s hematoxylin, and mounted.

Positive controls were represented by canine and human tissue samples from skin and liver biopsies exhibiting high parasite load. Negative controls used PBS followed by secondary antibody (normal mouse and rabbit serum, or negative serum from a mixed breed dog not infected with *Leishmania (L.) infantum*. The specificity of the primary antibodies was further determined by including canine tissue sections obtained from dogs not infected with *Leishmania (L.) infantum*.

The IHC slides were investigated with light microscopy for presence of amastigotes by three examiners. The slides were considered positive when at least two amastigotes of *Leishmania* were stained, with observations of size, shape, refringence, and location inside macrophages, as well as the presence of cytoplasmic staining of mononuclear inflammatory cells (particularly macrophages and epithelioid macrophages) and endothelial cells.

### IHC Labeling of *Leishmania* Amastigotes Using a Monoclonal *Leishmania* Antibody and Streptavidin-biotin Peroxidase

To assess the specificity of the standardized reaction using canine serum as a primary antibody, IHC assays were conducted employing a monoclonal antibody, anti-*Leishmania* lipophosphoglycan (Mo-anti LPG) clone CA7AE. This Mo-anti-LPG antibody recognized multiple Gal (β1,4) Man (α1)-PO4 repeat units of the LPG from many *Leishmania* species [18], [24].These repeat units are present in the proteophosphoglycans (PPGs) of amastigotes of *Leishmania*. We worked with two aliquots of antibodies: (1) a commercially available *Leishmania* lipophosphoglycan (LPG) monoclonal antibody obtained from CEDARLANE Laboratories, Canada, and (2) a laboratory-produced antibody following the method of Tolson et al. 1989 [25], gifted by Dr. Rodrigo Soares.

These monoclonal antibody aliquots, used as primary antibodies in parallel with the canine hyperimmune serum assays, were diluted 1∶100 in 0.01 M PBS. The detection protocol was as described above.

### IHC Labeling of *Leishmania* Amastigotes Using Dog Hyperimmune Serum as a Primary Antibody and Employing Biotin-free Polymer-based Technology

Since polymer-based technology is less time-consuming and labor intensive than the IHC labeled streptavidin-biotin peroxidase method, we compared the methods with all samples. Thus, a polymer-based non avidin-biotin method was carried out on all 73 human skin samples with LTA. These systems are usually 2-step procedures. The first step is the application of the unlabeled primary antibody; the second consists of the addition of a polymer containing secondary antibodies along with numerous enzyme molecules [26]. For the unlabeled primary antibody we used the same dilutions and conditions for the antibodies previously described: canine hyperimmune serum obtained from a dog naturally infected with *Leishmania (L.) infantum* and the monoclonal *Leishmania* lipophosphoglycan antibodies.

The first steps of this technique are similar to those described previously. After the addition of primary antibodies, slides were incubated at 37°C for 1 h in a humid chamber. Then, HRP ® Advance link was added to the slides, followed by the biotin-free polymer (DAKO HRP ® Advance - K 4068) and incubated for 30 min at room temperature. Slides were washed with PBS (2×5 min). The reaction was developed using 0.024% diaminobenzidine (DAB; Sigma, St. Louis, USA) solution and 0.16% hydrogen peroxide. Finally, the slides were dehydrated, cleared, and counter-stained with Harris’s hematoxylin.

### Statistical Analysis

The diagnostic tests (HE staining and IHC) were all calculated using Graph Pad Prism 5.0 software. The frequencies of positive results obtained from all samples were compared between test using the Person χ2 test with a 5% significance level and 95% confidence interval (IC) using the PCR as the gold standard. The Mann-Whitney test was used for comparison to numbers of amastigotes forms of *Leishmania*. In addition, the degree of agreement between the evaluated tests was determined by the coefficient Kappa (κ) values.

## Results

Seventy-three (73) patients with skin lesions suggestive of *Leishmania* infection were studied. Cutaneous lesions were observed in all patients who attended the reference center for leishmaniasis in Caratinga/MG. The criteria for being included in this study included a confirmed diagnosis of LCL based on the visualization of the parasite by smear and positive MST. Patients with LCL included 42 males and 31 females. The ages ranged from 1 to 78 years, average 33.82 years. The majority of patients were 31 to 45 years old. Sixty-nine patients (94.5%) lived in rural areas and four (5.5%) in urban areas, with one of those working in a rural area. Most were rural workers or farmers followed by non-agrarian occupations (homemaker). All patients showed ulcerous skin lesions typical of LCL, distributed as follows: 57 (78.1%) had single lesions, 11 had two lesions (15.07%), two (2.7%) had three lesions and three (4.1%) had four lesions. The average diameter of the 96 lesions was 18.90 mm. In general, lesions were reported on exposed body parts, with 39.6% on lower limbs, 37.5% on upper limbs, and 10.4% on the face. Lesions were also observed on the trunk (5.2%), abdomen (4.2%), and limb region (1.04%). From the patients’ medical records, it was determined that the time between the onset of the lesions and initial consultation ranged from 50 to 180 days with an average of 50.5 days. In 10 patients the time from onset of lesions to consultation was longer than 90 days.

**Figure 1:**
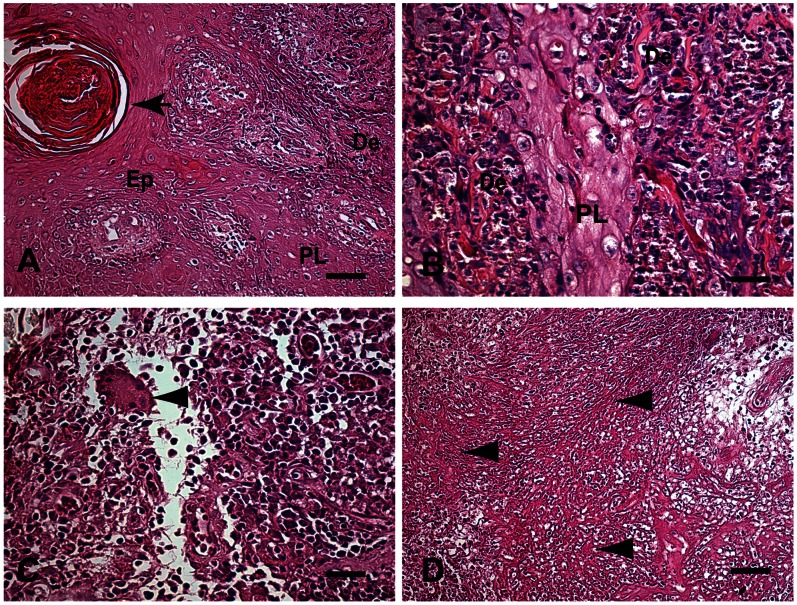
A–D: Fragment of skin of a patient with LCL Caratinga, MG, Brazil. (A) Changes observed in the epidermis were intense acanthosis (AC) and papillomatosis (PL). Pearl corneas can also be seen (black arrow). Finger-like projections of epidermis into the dermis layer, papillomatosis (PL) Bar = 32 µm, (B) Higher magnification shows thickening of the spinous (acanthosis) layer due to proliferation of epidermal cells (arrowhead) leading to papillomatosis (PL). Bar = 16 µm, (C) Higher magnification showing the inflammatory infiltrate of mononuclear cells (plasma cells, macrophages and lymphocytes) in the dermis. Note Langhans-type giant cell formation, but without a typical granuloma formation (arrowhead). Bar = 16 µm. (D) Eosinophilic necrotic area in the dermis with fragmented collagen fibers resembling fibrinoid necrosis (arrowheads) Bar = 64 µm. Hematoxylin-eosin staining. Epithelium (Ep), Dermis (De), Papillomatosis (PL).

The primary epidermal changes observed in the skin samples were: (1) moderate to intense hyperkeratosis and acanthosis (89.04%); (2) a slight to moderate papillomatosis (73.97%); (3) a slight to moderate parakeratosis (69.9%). Pearl corneas were formed in 43.8% of the cases. In 87.8% of cases, a moderate to severe chronic inflammatory reaction in the dermis characterized by a diffuse mononuclear exudate of plasma cells, macrophages and lymphocytes was observed (Figure 1A,B). We also identified *Langhans* giant cells and/or foreign body giant cell formation in 26.03% of cases (granulomatous chronic inflammatory reaction), but typical granuloma formations (exudative tuberculoid reaction) were not found Necrotic areas (necrotic fibrinoid) were observed in 41.1% of samples (Figure 1C,D). Fungal forms were not observed during HE examination. The PCR results were positive for all skin biopsies and it was the gold standard 73/73 (Table 1).

**Table 1:** Comparison among paired test HE, IHC (dog serum), IHC (LPG) and IHC (biotin-free polymer) according to result obtained by PCR in the diagnosis of ATL.

Positivity	Sensitivity (%)	Positivity PCR - 73/73 100%
HE 13/73	17.8 (%)	P<0.001
IHC (Dog serum) 67/73	91.8 (%)	P>0.05
IHC (Monoclonal LPG) 52/73	71.2 (%)	P<0.001
IHC (Dog serum biotin-free polymer) 67/73	91.8 (%)	P>0.05

P<0,05 - IC 95%.

**Figure 2:**
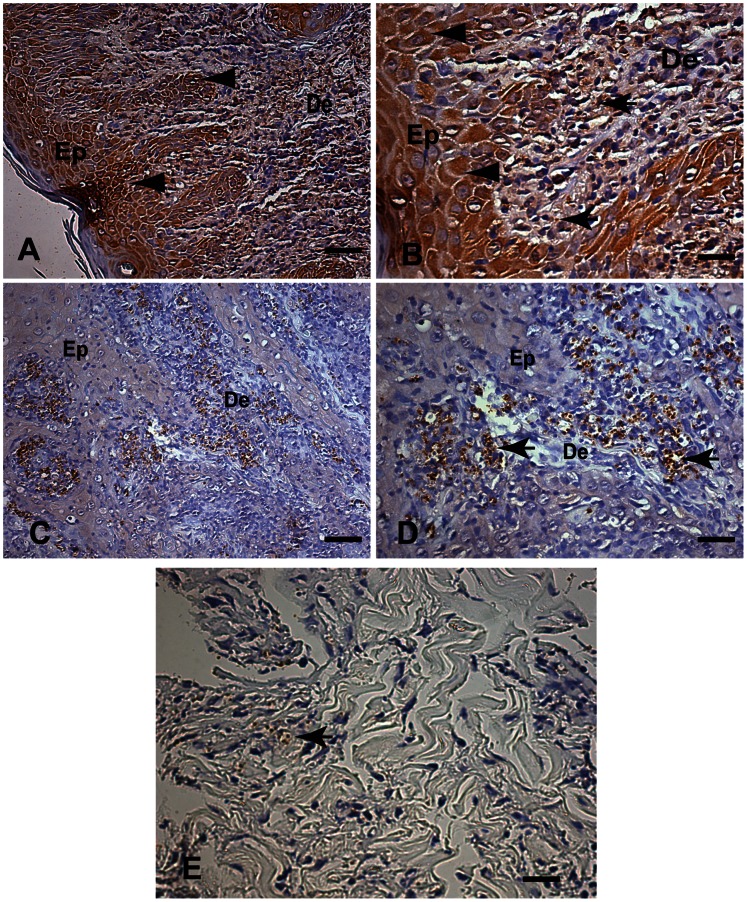
A–E: Fragment of skin of a patient with LCL, Caratinga, MG, Brazil. (A, B) Immunohistochemical labeling of amastigotes of *Leishmania* with an aliquot of a commercial monoclonal anti-*Leishmania* antibody and the streptavidin-biotin peroxidase method. (A) Low magnification showing a brown background evidenced by the cytoplasm of the epithelial layer cells (arrowheads). Bar = 32 µm. (B) Higher magnification showing intense non-specific staining visible as dark brown cytoplasmic staining of epithelial (arrowheads) and inflammatory mononuclear cells (macrophages) with intracellular amastigotes of *Leishmania* in the dermis (arrows) Bar = 16 µm. (C,D) Immunohistochemical labeling of amastigotes of *Leishmania* using dog hyperimmune serum as the primary antibody with the streptavidin-biotin peroxidase method (C) Low magnification showing light-blue stained background. Bar = 32 µm. (D) Higher magnification showing dark-brown-stained intracellular amastigotes of *Leishmania* within macrophages in the dermis (arrows) and light-blue-stained background. Bar = 16 µm; (A–D) Immunohistochemistry with the streptavidin peroxidase method counter-stained with Harris’s hematoxylin. (E) Observe immunolabeled amastigotes inside macrophages associated areas of tissue debris (arrow) Epithelium (Ep), Dermis (De).

Amastigote forms of *Leishmania* in histological sections stained with HE were visualized in 13 samples, corresponding to a sensitivity of 17.8% (13/73). The presence of some polymorphonuclear cells in the chronic exudate occurred in 28 (38.4%) of the samples analyzed. The positivity of the IHC method for labeling the amastigote forms of *Leishmania* with both different aliquots of the monoclonal *Leishmania* anti-LPG antibody using the streptavidin-biotin peroxidase method revealed the presence of amastigotes in 71.2% (52/73) of cases. We found intense non-specific dark-brown cytoplasmic staining of mononuclear cells and endothelial and epithelial cells (Figure 2A–B). In contrast, the IHC method for labeling amastigote forms of *Leishmania* exposed to the dog hyperimmune serum and developed by streptavidin-biotin peroxidase was 91.8% (67/73 cases). All HE positive cases were also positive by IHC. The degree of agreement between the evaluated tests was determined by calculating the coefficient Kappa (κ). The Kappa values showed no agreement between IHC and HE tests, fair agreement between IHC X LPG tests (0,2–0,39) and almost perfect agreement between IHC (with or without biotin-free polymer) X PCR tests (0,8–1).

The parasites were readily observed, especially within macrophages in the superficial and deep dermis. We believe this higher detection rate was due to the high contrast between dark brown stained amastigotes and the light blue stained background. Non-specific staining was not observed when the canine serum was used (Figure 2C,D). In fact, immunolabeled amastigotes were also find in cases of areas of tissue debris frequently hard to find by optical microscope (Figure 2E).

To determine the final dilutions for immune specific staining (*Leishmania*) and non-specific staining (background) we made dilutions for monoclonal *Leishmania* LPG antibodies and for the canine hyperimmune serum. Both monoclonal antibodies were serial diluted from 1∶100 to 1∶20,000 until amastigotes did not exhibit the dark brown staining. In parallel, canine hyperimmune serum was serially diluted from 1∶100 to 1∶40.000 until amastigotes did not exhibit the dark brown staining. However, we still observed epithelial cytoplasm nonspecific staining for both monoclonal LPG *Leishmania* antibodies. In contrast, we found immunolabeled *Leishmania* amastigotes in tissue without background staining with canine hyperimmune serum dilutions of 1∶10 to 1∶40,000.

**Figure 3:**
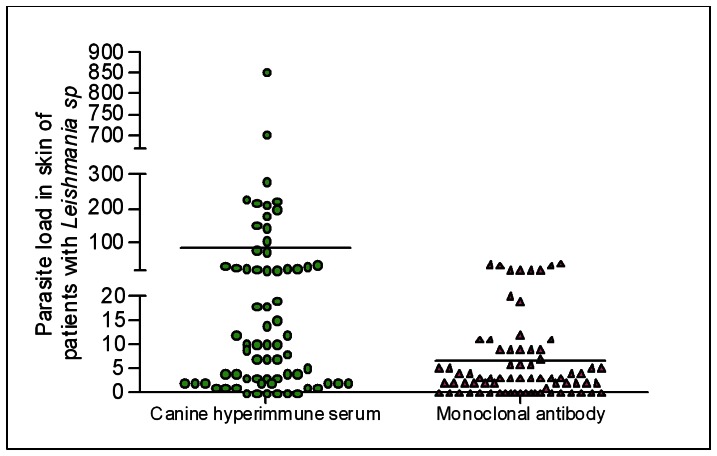
Parasite load of 73 skin fragments of a sample subjected to the immunohistochemical methods of streptavidin peroxidase, using hyperimmune serum from a dog naturally infected by *Leishmania infantum chagasi* (dilution 1∶100) and monoclonal antibody anti-*Leishmania* lipophosphoglycan (LPG) (Mo-anti LPG) (dilution 1∶100). Mann-Whitney p = 0.0001.

The number of *Leishmania* amastigotes identified by IHC using dog serum was significantly higher (p<0.05) than the number of amastigotes of the *Leishmania* parasite identified by IHC using both monoclonal *Leishmania* LPG antibodies (at a dilution of 1∶100 for all antibodies) (Figure 3). The skin parasite load of patients with LCL as determined by the IHC method using the dog serum was similar to that found with the monoclonal *Leishmania* LPG antibody (r = 0.2941).

The IHC method for labeling amastigote forms of *Leishmania* with dog hyperimmune serum using the biotin-free polymer-based technology showed the same sensitivity of 91.8% (67/73 cases) as the strepto-avidin-peroxidase system. Hence, the six skin samples that were negative for amastigotes using the dog serum and streptavidin were also negative using the biotin-free polymer system. In contrast to HE, IHC stained fungal forms as previously described.

## Discussion

Most of the ATL patients in the municipality of Caratinga, MG showed classical LCL lesions with a single lesion. These results are consistent with other ATL studies from Brazil [4], [27], [28] and other tropical countries. In fact, in Minas Gerais State, government records show the majority of cases to be LCL (95%) followed by MCL (3%) [29].

In the present study of ATL we did not propose or define histological profiles or classifications of skin lesions suggested by many authors [30]–[34]. However, the histology showed classical lesions previously described. Epidermal alterations frequently found were hyperkeratosis, acanthosis, and papillomatosis. In contrast to the epidermal changes described, the dermal lesions were similar among patients. In the majority of cases (87.7%), a chronic and diffuse inflammatory reaction was observed, with the cellular exudate mainly composed of plasma cells, macrophages, and lymphocytes. In 26.03% of these cases we also identified *Langhans* giant cells and/or foreign body giant cell formation, but without necrotic granuloma-like formations (unorganized granuloma). These results are consistent with histological patterns previously described as type I (exudative-cellular reaction without granuloma) and type IV (exudative-granulomatous reaction with unorganized granuloma) [32], [33]. Thus, a typical tuberculoid granuloma (organized granuloma) composed of epithelioid cells, with or without giant cell formations with central necrotic areas, was not found.

It is difficult to find parasites in active ATL lesions [34]. Lesions of patients with LCL are prone to heal after some months with or without treatment, resulting in smooth, shiny scars described as atrophic, hairless, and depressed, with hypo- or hyper-pigmented areas [31], [35], [36]. In the search for parasites in ATL lesions, the percent positivity is inversely proportional to the duration of infection [36], [37]. In ten patients the time of onset of lesions was greater than 90 days. The IHC method using the canine hyperimmune serum as the primary antibody with the biotin-avidin peroxidase complex or with polymer-based technology identified the presence of immunolabeled amastigotes in 100% of cases (10/10). The IHC method using the monoclonal antibodies was positive in70% of samples (7/10). This suggests another advantage of the proposed IHC method.

In the present study, 73 patients were diagnosed with ATL by three laboratory methods (smear, MST and PCR). After histological analysis we found parasites in only 13 skin samples (17.8%) from these patients. These results agree with many studies comparing the efficacy of diagnostic methods for ATL [9], [38]. Thus, our aim was to evaluate whether the IHC method proposed by Tafuri [23] is suitable for ATL diagnosis. Here, the IHC method for canine visceral leishmaniasis succeeded in detecting *Leishmania* in ATL patients. Sixty-seven skin samples (91.8%) of the 73 patients were positive for amastigote forms of *Leishmania*. In those positive samples, immunolabeled parasites were commonly observed in macrophages in the superficial and deep dermis. In contrast, the IHC labeled streptavidin-biotin peroxidase method using both *Leishmania* LPG antibodies showed a lower specificity than did the canine hyperimmune serum, with only 71.2% of the cases found positive. This could be explained lower detection due to the presence of non-specific background staining in all histological samples analyzed when stained with the commercial antibody.

Consistent with previous data [23], this study confirmed validity of immunohistochemical diagnostic techniques in skin biopsies from ATL patients. Since parasite concentrations in the lesions tend to be low, immunohistochemistry is useful as a supplementary tool for confirming a diagnosis based on HE staining. Some limitations of the IHC technique may be related to background reactions [8]. Our data showed that the pattern of immune labeled dark brown amastigotes with no background was low with IHC using dog hyperimmune serum in both streptavidin-peroxidase and the polymer-based methods. On the other hand, when using both LPG antibodies, the background reactions were higher. This may occur due to endogenous enzyme activity, inadequate fixation, sample degradation, or the presence of contaminating natural antibodies and proteins [8], [39]. The polymer-based technology produced results identical to the IHC method, with the advantage of being faster and less laborious.

Cross-reactions between the antibodies and non-target species of *Leishmania* suggest the possibility of using them in IHC to assess infection by different species and other trypanosomatids, including *Trypanosomacruzi* [23].This does not detract from the usefulness of the IHC method, since *T.cruzi* is found in host muscle tissue, rather than in skin [8], [40], [41]. We also detected cross-reaction between anti-*Leishmania* (*L*.) *infantum* hyperimmune serum and fungal forms (paracoccidioidomycosis) with the IHC method, but not in the HE slides (data not shown). Fungal infections are commonly found in the skin and can be clinically similar to leishmaniasis. Sporotrichosis, chromomycosis, paracoccidioidomycosis, and histoplasmosis have been reported [40]. However, histologically, the frequent presence of dermal necrosis associated with pseudo-epitheliomatous hyperplasia and intra-epidermal abscess formation, along with the identifiable fungal elements, differentiate these diseases. Here, IHC fungus-specific histochemical techniques (periodic acid-Schiff staining and Grocott’s silver impregnation) for all 73 ATL human skin biopsy samples were negative (data not shown).

In several ATL cases examined, occasional immunolabeled parasites masked by cellular and/or the matrix debris, or collagen fibrinoid necrosis, were difficult to identify by HE in skin samples. This finding is in accordance with Andrade-Narvaez *et al.* (2005) [42], working with ATL patients in Mexico, who showed that tissue changes, such as fibrinoid necrosis, could obscure infection and interfere with the reliability of the parasitological (histological) diagnosis. These authors found a lower parasite burden in chronic exudate, with or without granuloma formation that was associated with the presence of necrotic areas (tissue debris). Schubach *et al.* (2001) [43]described the possible use of IHC for identifying *Leishmania* antigens in tissues with partially degraded parasites or in the antigen-processing phase, independent of the visualization of any non-degraded amastigotes.

We have evaluated the effectiveness of an IHC protocol for the detection of *Leishmania* in human tissue with LCL in which canine hyperimmune serum was employed as a primary antibody. Indirect diagnosis was achieved in 91.8% of the patients studied. Both IHC labeled streptavidin-biotin peroxidase method and polymer-based technology were useful as supplementary tools to confirm the diagnosis based on HE stained sections. In addition, both IHC methods improved visualization of the amastigote forms of *Leishmania* via the use of a chromogenic substrate (DAB) at the antibody-antigen interaction site, which provided an excellent contrast with slight counterstaining and no nonspecific interactions. The polymer-based technology was less time-consuming than the IHC labeled streptavidin-biotin peroxidase method. These results suggest that further testing to validate this method for ATL diagnosis is justified.
